# Three-dimensional (3D) magnetic resonance volume assessment and loco-regional failure in anal cancer: early evaluation case-control study

**DOI:** 10.1186/s12885-020-07613-7

**Published:** 2020-11-30

**Authors:** Hema Sekhar, Rohit Kochhar, Bernadette Carrington, Thomas Kaye, Damian Tolan, Mark P. Saunders, Matthew Sperrin, David Sebag-Montefiore, Marcel van Herk, Andrew G. Renehan

**Affiliations:** 1 Division of Molecular & Clinical Cancer Sciences, School of Medical Sciences, Faculty of Biology, Medicine and Health, University of Manchester, Wilmslow Road, Manchester, M20 4BX UK; 2 Department of Radiology, The Christie NHS Foundation Trust, Manchester, UK; 3 Department of Clinical Radiology, St James’ University Hospital, Leeds, UK; 4 Department of Clinical Oncology, The Christie NHS Foundation Trust, Manchester, UK; 5 Division of Informatics, Imaging and Data Sciences, School of Health Sciences, Faculty of Biology, Medicine and Health, University of Manchester, Manchester, UK; 6 Leeds Institute of Cancer & Pathology, University of Leeds, St James’ University Hospital, Leeds, UK

**Keywords:** Anal cancer, Tumour volume, Loco-regional failure, Prognosis

## Abstract

**Background:**

The primary aim was to test the hypothesis that deriving pre-treatment 3D magnetic resonance tumour volume (mrTV) quantification improves performance characteristics for the prediction of loco-regional failure compared with standard maximal tumour diameter (1D) assessment in patients with squamous cell carcinoma of the anus undergoing chemoradiotherapy.

**Methods:**

We performed an early evaluation case-control study at two UK centres (2007–2014) in 39 patients with loco-regional failure (cases), and 41 patients disease-free at 3 years (controls). mrTV was determined using the summation of areas method (Vol_sum_). Reproducibility was assessed using intraclass concordance correlation (ICC) and Bland-Altman limits of agreements. We derived receiver operating curves using logistic regression models and expressed accuracy as area under the curve (ROC_AUC_).

**Results:**

The median time per patient for Vol_sum_ quantification was 7.00 (inter-quartile range, IQR: 0.57–12.48) minutes. Intra and inter-observer reproducibilities were generally good (ICCs from 0.79 to 0.89) but with wide limits of agreement (intra-observer: − 28 to 31%; inter-observer: − 28 to 46%). Median mrTVs were greater for cases (32.6 IQR: 21.5–53.1 cm^3^) than controls (9.9 IQR: 5.7–18.1 cm^3^, *p* < 0.0001). The ROC_AUC_ for mrT-size predicting loco-regional failure was 0.74 (95% CI: 0.63–0.85) improving to 0.82 (95% CI: 0.72–0.92) when replaced with mrTV (test for ROC differences, *p* = 0.024).

**Conclusion:**

Preliminary results suggest that the replacement of mrTV for mrT-size improves prediction of loco-regional failure after chemoradiotherapy for squamous cell carcinoma of the anus. However, mrTV calculation is time consuming and variation in its reproducibility are drawbacks with the current technology.

**Supplementary Information:**

The online version contains supplementary material available at 10.1186/s12885-020-07613-7.

## Background

Chemo-radiotherapy (CRT) is the primary treatment for patients with squamous cell carcinoma of the anus (SCCA) [1]. The key aim of treatment is to achieve loco-regional control whilst maintaining sphincter preservation. However, up to a quarter of those treated report loco-regional failure (LRF) within 3 years [2]. These patients may be offered a second attempt of cure with salvage surgery, but this is a radical operation associated with significant morbidity and poor long-term outcomes [3].

Until recently, CRT for SCCA adopted a broad “one size fits all” strategy, typically comprising 5-fluorouracil and mitomycin concurrent with 50 to 55 Gy radiotherapy, despite a wide spectrum of the loco-regional disease stages at presentation. Stratified approaches are now being evaluated, as exemplified by the UK PLATO (PersonaLisingrAdioTherapydOse in anal cancer, ISRCTN88455282) trial [4], a single protocol ‘platform’ comprising the ACT3, 4 and 5 trials with the aim of personalising radiotherapy dose across the disease spectrum. Presently, pre-treatment MR assessment of T-size (mrT-size), together with nodal status, are the key determinant of treatment stratification but this strategy has imperfections. For example, there is variation in maximum tumour dimensions determined using T2 weighting imaging [5]. One-dimensional tumour diameter may not adequately represent tumour biology that tends to be infiltrative in nature and irregularly shaped. Furthermore, based on using 7th edition AJCC staging (where the cut-off from T2 to T3 is 5 cm), Gunderson et al. [6] illustrated that there is a considerable ‘jump-up’ in 3-year LRF rates between T2N0 (10%) and T3N0 (22%), suggesting that T-size alone may not optimally capture the heterogeneity radio-resistance and risk of LRF.

An alternative imaging approach to pre-treatment staging is magnetic resonance quantification of tumour volume (mrTV). This has been explored in other tumour sites, such as in head and neck [7–10] and lung cancer [11–14]. However, methods and results have been inconsistent. Some studies use 3D quantification techniques while other have used semi-automated estimates of ellipsoid volume derived from 1D tumour dimensions. It is unclear whether this latter approach is a valid estimate.

The primary aim was to test the hypothesis that deriving pre-treatment 3D mrTV quantification improves performance characteristics for the prediction of LRF compared with standard maximal tumour diameter (1D) assessment in patients with SCCA undergoing CRT. We chose to quantify 3D mrTV using a summation of areas method, as conceptually, this better captures the complex 3-dimensional nature of an anal tumour compared with estimation methods derived from 1D tumour dimensions. But in turn, this method may be labour intensive and there may be considerable variation in reproducibility. Thus, secondary aims were to evaluate whether 3D mrTV quantification directly measured on scans by the summation of area method is reproducible; and whether volume estimation derived from less labour intensive 1D tumour dimensions is a valid estimation of tumour volume.

## Methods

In accordance with the CRUK/EORTC imaging biomarker consensus statement, this was a two-centre Domain 2 validation study evaluating performance characteristics, reproducibility and whether the biomarker is ‘fit for purpose’ [15].

### Patients and treatment

We performed a case-control study at two UK centres, the Christie NHS Foundation Trust, Manchester, and Leeds Teaching Hospitals Trust, Leeds (LTHT). Patients were included if they had histologically confirmed SCCA; T1 to T4 disease (AJCC 7th edition) [16]; and had received CRT with curative intent for non-metastatic disease. For the control group, patients were free of LRF for at least 3 years follow-up. Patients with histologies other than SCC were excluded, as were patients where T-stage was undeterminable (Tx disease,) as mrTV and mrT-size parameters could not be quantified.

All patients were treated between 2007 and 2014 prior to the introduction of IMRT (Intensity Modulated Radiotherapy). The treatment protocol followed that used in the ACT II trial [17] – namely, radiotherapy of 50·4 Gy was delivered over 5·5 weeks with a two phase technique, without a mandatory break. Phase 1 included 30·6Gy in 17 daily fractions with non-conformal rectangular parallel-opposed fields. Phase 2 required conformal planning and delivered 19·8Gy in 11 daily fractions over 15 days to the primary tumour with a 3 cm margin and any involved lymph nodes. Chemotherapy regimens were administered concurrently with radiotherapy as either: mitomycin-C (MMC) 12 mg/m^2^ on day 1, and continuous infusion of 5-fluorouracil (5-FU) 1000 mg/m^2^ on days 1–4 and days 29–32.

### Selection for case-control study

From the retrospective two-centre clinical databases, all 40 patients with LRF from 2007 to 2014 undergoing CRT and with measureable anal tumours were selected as cases (one outlier volume later excluded). Forty-one patients without LRF at 3 years were controls. Control selection was at random from those patients in the databases with available MR images satisfying criteria listed next.

### Tumour volume quantification

All tumour quantification used routinely collected pre-treatment MR imaging, performed on a 1.5 Telsa MR employing optimal pelvic phase-array body coil (acquisition protocols are detailed in the supplementary material, Table S1). For inclusion, scans had to meet the following criteria: (i) include a small field of view high resolution T2-weighted (T2W) sequence in the axial plane as minimum, with a slice thickness ≤ 4 mm; (ii) field of view extending above and below the tumour in two orthogonal planes to allow complete tumour assessment including TV quantification and assessment of T-size; and (iii) where the tumour required more than one series to assess the whole tumour, then these series had to overlap sufficiently such that the entire tumour was imaged and could be quantifiable.

To determine mrT-size, three primary orthogonal measurements of the maximal diameters were taken in the anterior-posterior *(AP)*, left-right *(LR)* and cranio-caudal *(CC)* planes along the axis of the tumour measured on the high resolution T2W images. The *AP* and *LR* diameters were recorded on the axial plane at the point of maximal dimension. The longest diameter was noted and the next dimension was taken at an axis perpendicular to the above. *CC* dimension was measured in either the coronal or sagittal plane. The largest of these three diameters was considered to be the tumour size (cm). Assessors (RK and BC) were blind to LRF status.

mrTV measurements were performed using World-Match (in-house written software from MvH [18]) that allowed simultaneous contouring on several sequences of different planes. Pre-treatment MR images were imported in anonymised DICOM format and the primary tumour was manually contoured. Delineations were checked and adjusted accordingly using coronal and sagittal planes. All contiguous areas of tumour were contoured together including nodal masses that had coalesced with the tumour or contiguous areas of extra-mural vascular invasion (EMVI). This was required to account for difficulties in defining a plane between the entities and to allow consistency of approach. Separate or discrete nodal volumes were not included. The assessor (HS) was blind to LRF status.

**Fig. 1:**
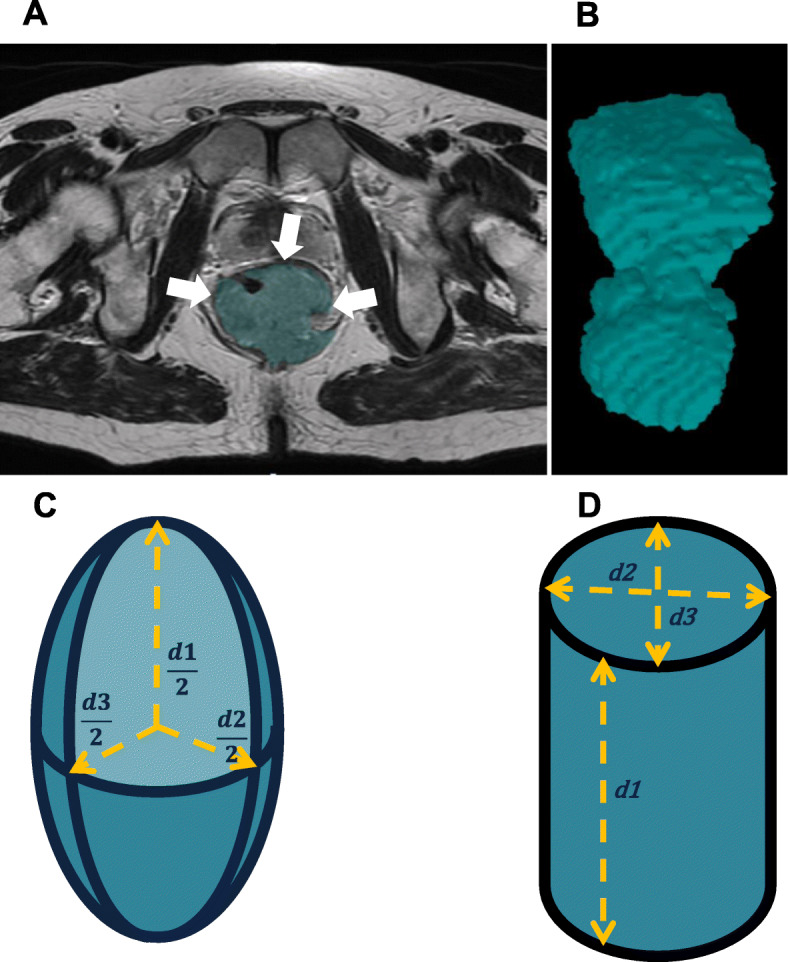
Method of quantification of tumour volume: **a** pelvic axial section of MR images depicting an anal canal tumour contoured in blue (arrows); **b** sequential contours rendered to create 3D representation of the tumour, demonstrating its irregular and complex shape; and **c** estimation of volume using orthogonal tumour size measurements under the assumption that the tumour volume approximates an ellipsoid. **d** estimation of volume using orthogonal tumour size measurements under the assumption that the tumour volume approximates an elliptical cylinder. Figures (**a**) and (**b**) were created using World-Match (inhouse written software). Figures (**c**) and (**d**) were created using Microsoft Office Powerpoint

For the main analysis, mrTV was derived by using a summation of areas method (Vol_sum_), which sums-up the area contoured on serial image slices while taking into account the distance between the slices; i.e. the slice thickness of the scans (Fig. 1a & b). The time taken to contour the TV on MR images, was recorded for the first 22 patients (‘training’) and compared with the remaining patients.

### Intra-and inter-observer variability

Intra- and inter-observer variability of mrTV quantification was assessed in ten (randomly selected) patients and compared using intra-class concordance correlations (ICC) with scores < 0.5 representing poor; 0.5 to < 0.75 moderate; 0.75 to < 0.9 good; and ≥ 0.9 excellent agreement [19]. 95% confidence intervals were derived from z-transformations. Bland-Altman plots were employed to further assess agreement [20]. We standardised all plots so that y-axis (mean difference between measure modalities) ranges were equivalent to those of the x-axis (mean value from both measure modalities). We then examined each plot for: (i) mean values closeness to zero; (ii) levels of agreement; and (iii) that the pattern across the range of means was proportionate i.e. evaluating for trends across the range. For levels of agreement, we expressed as percentage of the x-axis range of values and reported as these as ‘wide’ if the limits fell outside +/− 10% of the average mean difference.

### Ellipsoid and elliptical cylinder volume estimation

We tested whether volume estimation derived from 1D tumour dimensions is a valid estimate of TV, using ellipsoid and elliptical cylinder equations and measured 1D diameters (Fig. 1c & d), where d1, d2 and d3 are the maximal diameters of the primary tumour measured in the *AP, LR* and *CC* planes. The ellipsoid equation was: $\frac{4}{3}\times \pi \times \frac{d1}{2}\times \frac{d2}{2}\times \frac{d3}{2}$; the elliptical cylinder equation was: (*π* × *d*1 × *d*2 × *d*3)/4. These were assessed for reproducibility as above. We also evaluated for accuracies compared with Vol_sum_ using the ROC_AUC_ method (we expected ellipsoid and elliptical to be equivalent as they are derived from the same parameters).

### Statistical analysis

Stata software, version 14 (Stata Corp., Tx, USA) was used for all statistical analyses. Continuous data were summarised as medians with inter-quartile ranges (IQR) and categorical data were presented as proportions. Comparisons were by Chi-square and Fisher’s Exact tests and non-parametric Mann-Whitney U tests, respectively.

Assessment of the discriminatory potential of the different tumour quantifications was tested with receiver operator characteristic curves (ROCs) and estimation of accuracy using the AUC and compared against each other using the method of DeLong et al. [21]. Multivariable models used logistic regression, and derived ROC_AUC_ using post-estimation commands.

In our power calculation, we posited that 10% would be a meaningful clinical difference for areas under the curve ROC_AUC_. We added 3% as case-control studies tend to overestimate performance characteristics [22]. Thus, we concluded that 42 cases of LRF and 42 controls of non-LRF patients would be required to reach ROC_AUC_ difference of 13% at *α* < 0.001.

We utilised other indicators of performance characteristics, using the methods described by Pencina et al. [23] which derives two characteristics – the Integrated Discriminatory Improvement (IDI), an index of improvements in sensitivity relative to specificity, and Net Reclassification Improvement (NRI), an index of net change in events versus non-events detected, which in turn focuses on medical decision making.

## Results

### Patient characteristics

Eighty-one patients were initially included in the study. One case with a tumour volume of 652 cm^3^ was excluded as an extreme outlier, leaving 80 patients as characterised in Table 1. Sixty-one patients were treated at The Christie and 19 at LTHT. The cases and controls were well-balanced for age and gender. As expected, the LRF group had more patients presenting with T3/4 disease compared with the non-LRF group and more patients presenting with node positivity. There were no differences noted in these baseline variables between the patients from The Christie and LTHT (Table S2).

**Table 1:** Patient characteristics of those with and without locoregional failure

	Controls Non-LRF	Cases LRF	***p*** value
**N**	41	39	
**Gender**			
Men (%)	14 (34)	16 (41)	0.525^a^
Women (%)	27 (66)	23 (59)	
**Median age (IQR), years**	60 (53–69)	57 (50–69)	0.563^b^
**mrT-stage (%)**			0.018^c^
T1	1 (2)	0	
T2	24 (59)	11 (28)	
T3	9 (22)	16 (41)	
T4	7 (17)	12 (31)	
**mr Nodal status**			
LN^−^ (%)	26 (63)	14 (36)	0.014^a^
LN^+^ (%)	15 (37)	25 (64)	

*LRF* Locoregional Failure, *non-LRF* Without evidence of locoregional failure after 3-years follow-up, *IQR* Interquartile Range, *LN+* Nodal Involvement; ^a^Chi-square test; ^b^Mann-Whitney U Test; ^c^Fisher’s Exact Test

### Volume quantification

mrTV measurements took a median of 7 min, but with wide variation from less than 1 min to over an hour. Contours performed in the 22 preliminary patients took longer than subsequent determinations [median: 10.5 (IQR: 7.2–14.3) minutes versus 5.6 (IQR: 4.3–12.2) minutes, *p* = 0.018].

The median volume in all 80 patients determined by summation of areas was 20.1 (IQR: 9.1–39.0) cm^3^. The median mrTV increased with mrT-stage as follows: T1, 5.7 cm^3^; T2, 9.1 (IQR: 4.6–16.7) cm^3^; T3, 38.4 (IQR: 21.6–50.2) cm^3^; T4, 34.5 (IQR: 18.1–66.5) cm^3^, *p* < 0.001 (Figure S1).

### Reproducibility

**Fig. 2:**
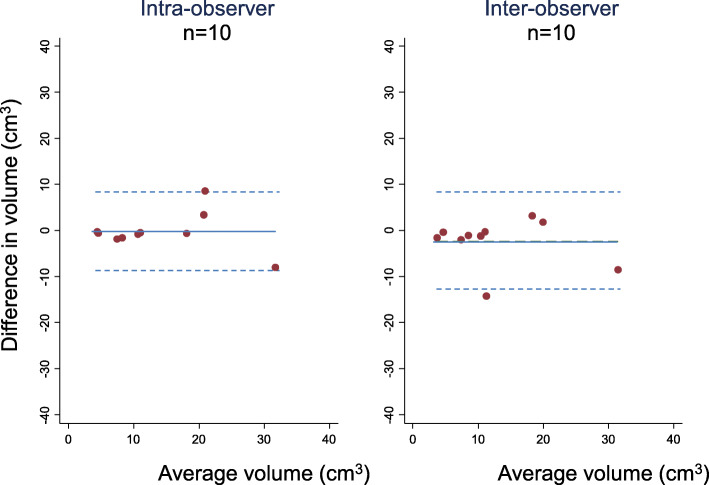
Bland-Altman plots demonstrating intra- and inter-observer (modest) agreement of tumour volume quantification in 10 patients: **a** average mean difference of − 0.27 cm^3^ and limits of agreement from − 7.93 cm^3^ (− 28%) to 8.47 cm3 (31%) for intra-observer variability; and **b** average mean difference − 2.5 cm3 and limits of agreement − 7.65 cm^3^ (− 28%) to 12.59 cm^3^ (46%) for inter-observer variability. Figures were created using Stata software, version 14

Reproducibility was generally good. Intra-observer ICC scores were 0.89 (95% CI: 0.75 to 1.00); inter-observer ICC scores were 0.79 (95% CI: 0.55 to 1.00). Agreement was further explored with Bland-Altman plots, which revealed wide limits of agreement from − 7.93 cm^3^ (− 28%) to 8.47 cm^3^ (31%) for intra-observer variability and − 7.65 cm^3^ (− 28%) to 12.59 cm^3^ (46%) for inter-observer variability (Fig. 2).

### Tumour size versus volume

**Fig. 3:**
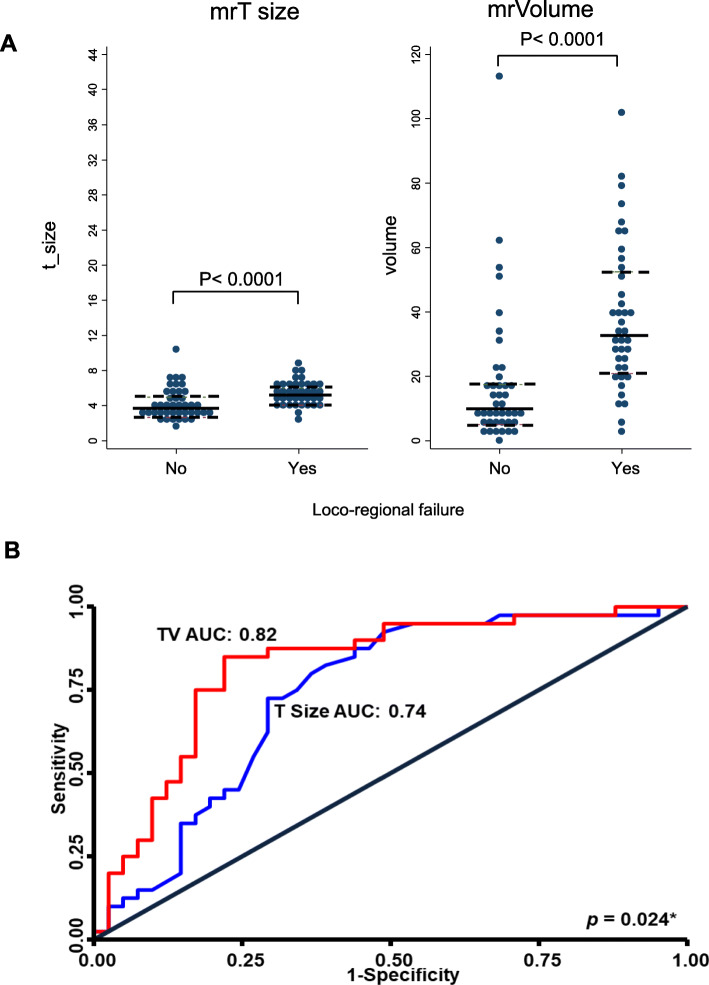
**a** Dotplots demonstrating the association of tumour volume and T-size with loco-regional failure (LRF) with the patient with the large volume outlier removed from both analyses: The horizontal black lines represent the median tumour quantification values; the dashed black lines represent the limits of the interquartile range. The y-axis has been standardised so that the medians in the controls are aligned (**b**): Receiver Operator Characteristic curves comparing the discriminatory performance of measured tumour volume and mrT-size. ^*^AUCs compared by the method of DeLong et al. Figures were created using Stata software, version 14

Median mrT-size was larger in cases compared with controls [5.2 (IQR: 4.3–6.2) cm versus 3.6 (IQR: 2.9–5.2) cm, *p* = 0.0004]. Similarly, mrTV determined by summation of areas was greater in cases compared with controls [32.6 (IQR: 21.5–53.1) cm^3^ versus 9.9 (IQR: 5.7–18.1) cm^3^, *p* = 0.0001] (Fig. 3a).

The ROC_AUC_ for mrT-size predicting LRF was 0.74 (95% CI: 0.63–0.85).This improved to 0.82 (95% CI: 0.72–0.92) when mrTV replaced mrT-size (*p* = 0.024) (Fig. 3b). As nodal status is a predictor for LRF [2], we adjusted for this in two separate multivariable logistic regression models for mrT and mrTV. The ROC_AUC_ did not materially change (Table 2).

**Table 2:** Univariable and multivariable AUCs from ROCs for outcome of loco-regional failure (*N* = 80)

	Univariable	Multivariable^a^
Variable	AUC (95% CI)	AUC
**Gender**	0.534 (0.427–0.642)	
**Age (years)**	0.463 (0.333–0.592)	
**T-Size (cm)**	0.731 (0.617–0.846)	0.738^b^
**Tumour Volume (cm**^**3**^**)**	0.817 (0.720–0.915)	0.801^b^
**Nodal status**	0.638 (0.531–0.744)	

*CI* Confidence Interval

^a^These are two variable models – the variable listed in the left-hand column plus nodal status

^b^ No 95% CIs are returnable in these post-estimations

### Ellipsoid and elliptical cylinder estimates

The median volumes estimated from the ellipsoid equation was 20.6 (IQR: 8.6–44.7) cm^3^. That for volume estimated from elliptical cylinder equation was higher at 30.8 (IQR: 13.0–67.0) cm^3^. Agreement was excellent between Vol_sum_ and volume determined by the ellipsoid eq. [ICC scores =0.91 (95% CI: 0.86 to 0.94)] and good between Vol_sum_ and volume determined by the elliptical cylinder eq. [ICC scores =0.73 (95% CI: 0.66 to 0.80)]. From, the Bland-Altman plots, the limits of agreement for Vol_sum_ and volume determined by the ellipsoid equation were from − 20.4 (− 16%) to 20.1 (17%). For Vol_sum_ versus volume determined by the elliptical cylinder, there was a disproportionate trend with increasing volume (Figure S2).

The ROC_AUC_ for mrTV determined by the ellipsoid volume estimate was 0.79 (95% CI: 0.68 to 0.89); that for the elliptical cylinder volume estimate was identical (Table S3).

### IDI and NRI

For mrTV (measured as Vol_sum_) versus mrT-size, the IDI was 9.6% (standard error, se: 3.0%), which was statistically different (*p* = 0.0015). By contrast, the NRI was potentially large at 34% (se: 22%), but not statistically significant (*p* = 0.123).

## Discussion

### Summary of Main findings

mrTV quantification determined by summation of areas may improve accuracy to predict for loco-regional failure when replacing mrT-size. However, this method is time consuming and variation in reproducibility are drawbacks with current technology. The use of tumour volume estimates that utilise routinely obtained 1D tumour diameter measurements (ellipsoid and elliptical cylinder equations) had imperfect levels of agreement with volume determined by summation of areas but it is unknown whether this influences performance characteristics defined by AUC on ROC analyses.

### Context of other literature

To our knowledge, this is the first study to evaluate the performance characteristics of mrTV and LRF in SCCA. Many studies [24–29] in patients with SCCA have reported on TNM-related parameters as treatment predictors. Of the six published trials in this field, three [30–32] reported tumour predictive information, using either AJCC T-stage or a single one-dimension tumour size cut-off. In contrast, tumour biology occurs at a 3D level, with the chance of cure being dependent on factors such as increasing number of tumour clonogens that require sterilising and the extent of tumour hypoxia, both of which increase with the bulk of the tumour. Thus, tumour volume seems a ‘closer to real-life’ model for tumour biology.

MR imaging is the current standard of care for the pre-treatment staging of SCCA [33], providing high-resolution imaging. It is tempting to speculate that extension of this imaging platform to tumour volume quantification could improve treatment prediction. MR imaging additionally allows analysis of tumour heterogeneity, another parameter that may be a better representation of tumour biology and offer complementary information to current staging parameters. One study of forty patients [34] found tumour heterogeneity is associated with disease recurrence.

### Limitations and strengths

The study has several strengths. First, we used data from two well-characterised UK treatment centres with uniform treatment protocols. Second, to minimise the risk of heterogeneity in scanning parameters and quality over time and between centres, scan quality parameters were evaluated. Third, in-house written software was employed, which allowed contouring in multiple planes, facilitating accurate volume quantification.

The study has several limitations. First, with only 80 patients, it is a relatively small sample size. Second, to enrich for events we used a case-control study design. This approach is biased in that it overestimates performance characteristics [22] and hence we powered using a very conservative *α* < 0.001. Third, we fell just short of our target sample size. An improvement of 9% in the ROC_AUC_ for mrTV over mrT-size was statistically significant at *p* = 0.024, and might be clinically meaningful. A similar 9% was noted with the IDI method, and while the NRI was potentially promising at 34%, it had much uncertainty. Fourth, as a retrospective study, there are potential biases and confounding. To mitigate against some of these, the assessors were blinded. Finally, the study recognises that ‘drawing’ tumour volume on MR is currently imprecise. It is challenging to differentiate peri-tumour oedema and fibrosis from tumour tissue and pathological validation of TV measurements was not possible. Indeed, tumour delineation is one of the most uncertain aspects of RT planning and as such, assessment of tumour volume for the purpose of prediction is subject to the same issues [35–39]. This is reflected in the moderate-good intra- and inter-observer concordance. In a parallel set of data (unpublished), we found there is modest intra- and inter-observer variability for T-size (ICC for intra-observer variability: 0.86; ICCs for inter-observer variability ranging from 0.73 to 0.82).

### Clinical implications and future research

The findings from this study suggest that 3D MR-based tumour volume quantification may enhance prediction of LRF risk over current MR-based methods. We speculate that TV better reflects tumour biology and could facilitate risk stratification to improve the precision of personalised treatment. The main limitation is that tumour ROI definition on MR is not currently routinely performed, and can be time consuming. MR-based RT planning, currently investigated in tumours such as rectal adenocarcinoma [40], may become routine in the future for SCCA, requiring tumour delineation for treatment planning which can then be reused as a prognostic factor. Techniques for automated contouring may also offer a time-sparing solution in the future, once developed and validated. Our hypothesis must now be evaluated – for example by, ‘piggy-backing’ mrTV definition onto ongoing trials.

Tumour regression grading has been found to predict for early local failure in SCCA [41]. Further work examining the role of measured tumour volume reduction following CRT may provide more prognostic information; however, differentiating tumour from fibrosis and oedema following CRT is a significant challenge.

PET-CT is increasingly used in the pre-treatment assessment of anal cancer and small studies demonstrate the potential prognostic role of metabolic tumour volume (MTV) [42–45]. Large scale exploration of the prognostic role of MTV could be useful, and since PET-CT does not necessarily detect areas of tumour necrosis [46], which are unlikely to contain clonogenic cells, an important factor in the probability of cure, examining the predictive role of MTV relative to TV would be of interest.

Lastly, we acknowledge that looking at a single parameter to predict LRF is simplistic and it is more realistic that a number of patient, biological, tumour and radiological features each contribute to prognostic outcomes in anal cancer [47], and are likely to be incorporated into future risk-adapted treatment strategies. A multiparametric prognostic model incorporating all of these features would be ideal, but investigating this issue is outside of the remit of this preliminary study. Larger-scale studies that facilitate this and are in progress. We have, however, identified mrTV to be a potentially useful prognostic tool, to be studied within such future models.

## Conclusions

These preliminary results suggest that 3D MR-based tumour volume quantification may improve prediction of LRF in patients with SCCA following CRT over currently employed measurements. Further work will be required to refine ROI contouring, both in regards to facilitating a less time consuming and more reproducible process, and to explore the role of this tumour volume within multiparametric prognostic models for LRF in SCAA.

## Supplementary Information

**Additional file 1.**

## Data Availability

The datasets supporting this article are stored in a secured research database and may be available upon presentation of formal approval.

## Abbreviations

3D: Three Dimensional
5-FU: 5-Fluorouracil
AJCC: American Joint Committee on Cancer
AP: Anterior-posterior
AUC: Area Under the Curve
CC: Cranio-caudal
CI: Confidence Interval
CM: Centimetre
CRT: Chemoradiotherapy
CRUK: Cancer Research United Kingdom
DICOM: Digital Imaging and Communications in Medicine
EMVI: Extra-mural Vascular Invasion
EORTC: European Organisation for Research and Treatment of Cancer
Gy: Gray
ICC: Intraclass Concordance Correlation
IDI: Integrated Discriminatory Improvement
IMRT: Intensity Modulated Radiotherapy
IQR: Inter-quartile Range
LR: Left-right
LRF: Loco-regional Failure
LTHT: Leeds Teaching Hospitals Trust
MMC: Mitomycin-C
MR: Magnetic Resonance
MTV: Metabolic Tumour Volume
NHS: National Health Service
NRI: Net Reclassification Improvement
PET-CT: Positron Emission Tomography - Computed Tomography
PLATO: PersonaLisingrAdioTherapydOse in anal cancer
ROC: Receiver Operator Characteristic
ROI: Region Of Interest
RT: Radiotherapy
SCCA: Squamous Cell Carcinoma of the Anus
se: Standard Errors
T2W: T2-weighted
TNM: Tumour-Nodal-Metastases
TV: Tumour Volume
UK: United Kingdom
VOLsum: Volume from summation of areas
