# Supplementary material for: Three-dimensional (3D) magnetic resonance volume assessment and loco-regional failure in anal cancer: early evaluation case-control study
Source: BMC Cancer. 2020 Nov 30;20:1165. doi: 10.1186/s12885-020-07613-7 (PMC7706015; doi:10.1186/s12885-020-07613-7)

**Supplementary Material**

**Three-Dimensional (3D) Magnetic Resonance Volume Assessment and Loco-regional Failure in Anal Cancer: early evaluation case-control study**

**Sekhar et al.**

**Table S1 MR Acquisition protocols for both Christie and Leeds**

|  | **Sequences** | **Acquisition Parameters** | **Comment** |
| --- | --- | --- | --- |
| Tumour | HR^1^ T2 sagittal TSE | TR/TE 5390/100 ms; NEX 3; ST 3 mm; FOV 200 | Sacral promontory cover anal margin |
| Tumour | HR^1^ T2 axial TSE | TR/TE 5030/100 ms; NEX 2; ST 3 mm; FOV 200 | Perpendicular to long axis of anal canal, x 2 overlapping blocks |
| Tumour | HR^1^ T2 coronal TSE | TR/TE 6500/137 ms; NEX 2; ST 3 mm; FOV 200 | Parallel to long axis of anal canal |
| Tumour | Diffusion axial T2-weighted SS-EPI | TR/TE 3900/ 89 ms; NEX 5; ST 5 mm; FOV 320 | b values 50,400,800,1000 |
| Tumour | STIR axial  STIR coronal / sagittal | TR/TE 9840/97 ms; NEX 1; ST 3mm  TR/TE 5960/68 ms; NEX 2; ST 3mm | Only if suspicion of fistula |
| Abdomen / Pelvis | T1 axial SE | TR/TE 400/12 ms; NEX 1, ST 5mm; FOV 380 | X 2 overlapping blocks, renal hila to symphysis |
| Abdomen /pelvis | T1 coronal SE | TR/TE 668/19 ms; NEX 2, ST 6mm; FOV 490 | Diaphragm to symphysis pubis |

HR: High-Resolution; TSE: Turbo Spin Echo; TR: Repetition Time; TE: Echo Time; NEX: Number of Excitations; ST: Slice Thickness; FOV; Field of View; SS-EPI: Single Shot Echo Planar Imaging; STIR: Short Tau Inversion Recovery Sequence.

**Table S2 Patient Characteristics stratified by sites**

|  | **Site 1** | **Site 2** | ***p* value** |
| --- | --- | --- | --- |
| **N** | 61 | 19 |  |
| **Gender** |  |  |  |
| Men (%) | 25 (41) | 5 (26) | 0.249* |
| Women (%) | 36 (59) | 14 (74) |  |
| **Median age (IQR), years** | 60 (51-69) | 55 (48-64) | 0.2417† |
| **mrT-stage (%)** |  |  | 1.000‡ |
| T1 | 1 | 0 |  |
| T2 | 27 (44) | 8 (41) |  |
| T3 | 19 (31) | 6 (32) |  |
| T4 | 14 (23) | 5 (26) |  |
| **mr Nodal status** |  |  |  |
| LN^-^ (%) | 30 (49) | 10 (53) | 0.793* |
| LN^+^ (%) | 31 (51) | 9 (47) |  |

IQR:Interquartile Range; LN+: Nodal Involvement; *Chi-square test; †Mann-Whitney U Test; ‡Fisher’s Exact Test.


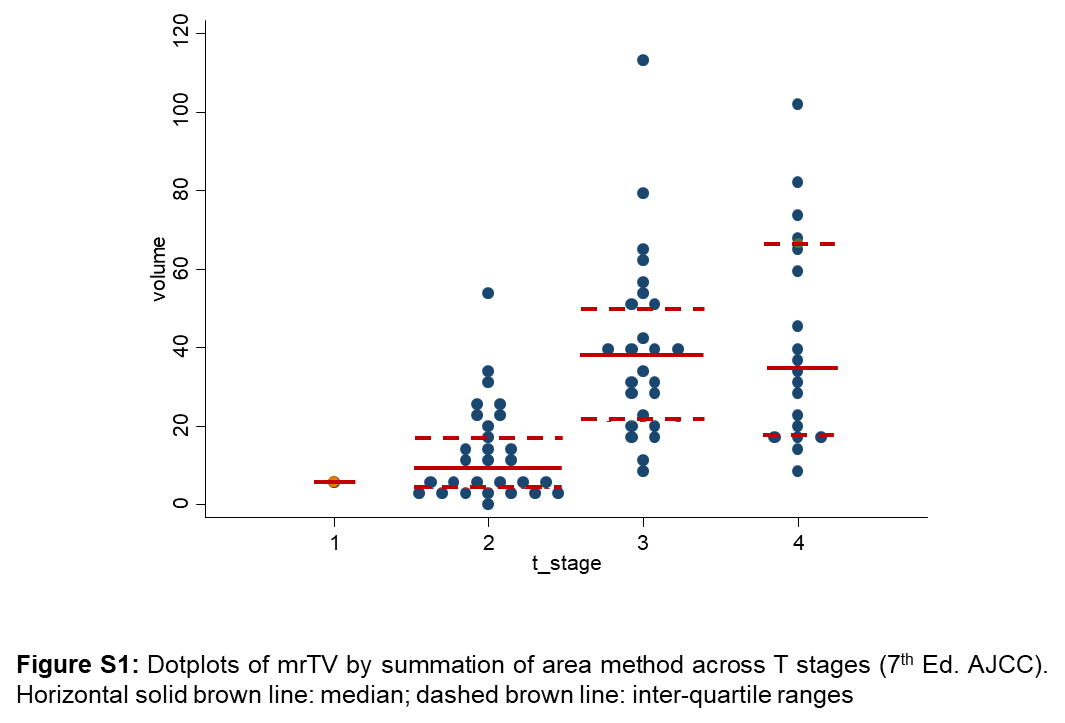


**Table S3 AUCs from ROCs for outcome of loco-regional failure (N = 80) by different measures of tumour volume (all models are unadjusted)**

|  | **Median volume**  **(IQR)**  **(cm^3^)** | **Univariable** |
| --- | --- | --- |
| **Variable** |  | **AUC (95% CI)** |
| Tumour volume by summation of areas | 20.1  (9.1-39.0) | 0.817  (0.720-0.915) |
| Tumour volume by ellipsoid equation | 20.6  (8.6-44.7) | 0.785  (0.682-0.888) |
| Tumour volume by elliptical cylinder equation | 30.8  (13.0-67.0) | 0.785  (0.682-0.888) |

CI: Confidence Interval


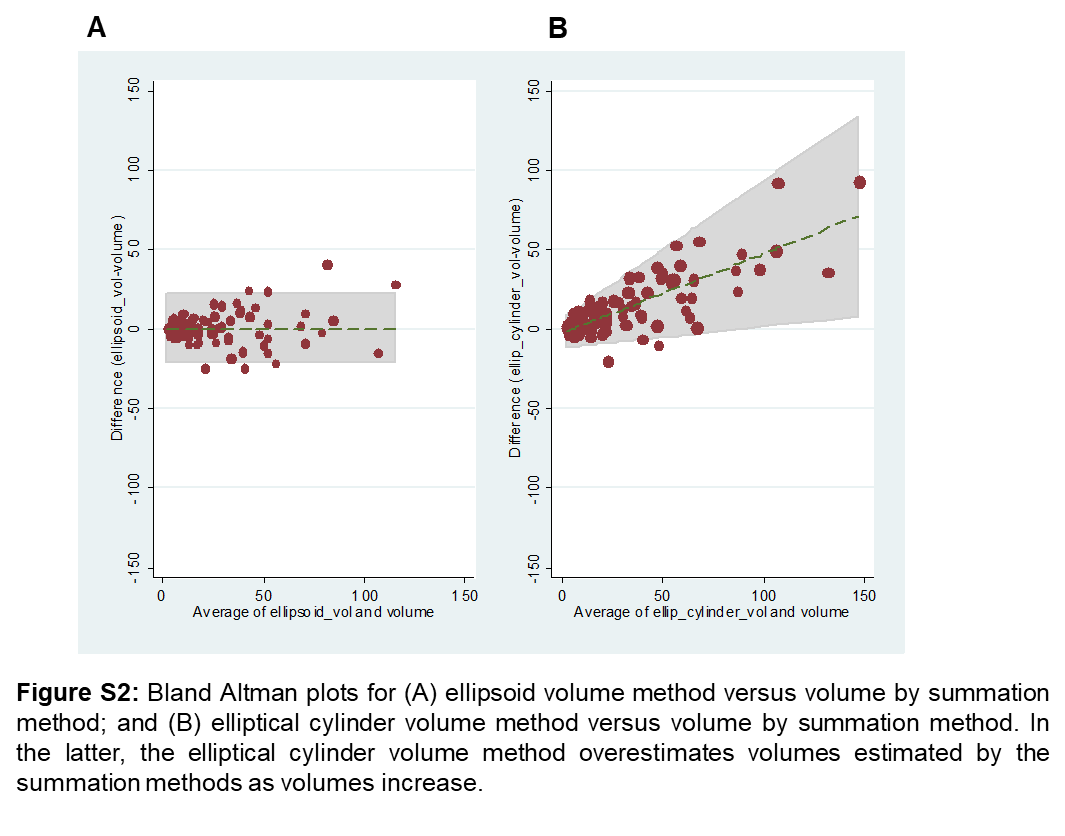

Supplement: Supplementary file 1 — Additional file 1. [file 12885_2020_7613_MOESM1_ESM.docx]
